# Delayed Diagnosis of Retained Surgical Blade 12 Years Post-Hysterectomy: A Rare Case Report

**DOI:** 10.1155/cris/8620883

**Published:** 2025-10-23

**Authors:** Alfred Kishe, Agathon Avelin Kimario, Ronaldo Paul Lyimo, Nancy Deliko Ngaga, Joel Pilot Mushi, Emmanuel Pastory Marua

**Affiliations:** ^1^Department of Surgery, St. Joseph Council Designated Hospital, Moshi P.O Box 330, Kilimanjaro, Tanzania; ^2^School of Medicine, Kilimanjaro Christian Medical University College (KCMUCo), Moshi P.O Box 2240, Kilimanjaro, Tanzania

**Keywords:** case report, delayed diagnosis, hysterectomy complication, retained surgical instrument, surgical safety

## Abstract

**Introduction:**

Retained surgical instruments (RSIs) are rare but serious surgical complications. This report presents a unique case of a retained surgical blade identified 12 years post-hysterectomy, highlighting diagnostic challenges and the need for vigilance.

**Case Presentation:**

A 60-year-old female presented with chronic abdominal pain for 4 years, initially misdiagnosed as urinary tract infection (UTI) and gastritis. Investigations, including X-ray and computed tomography scan (CT scan), revealed a retained surgical blade. Elective laparotomy was performed, and the rusted blade, encapsulated by the omentum, was removed. Postoperative recovery was uneventful.

**Discussion:**

The delayed diagnosis underscores vulnerabilities in surgical safety protocols. Nonspecific symptoms of RSIs often lead to delayed detection. While manual counting is the standard, human error can occur. This case emphasizes the need for advanced technologies and standardized protocols. Underreporting of RSIs obscures true rates, necessitating improved data transparency and systemic learning.

**Conclusion:**

This case highlights the importance of multidisciplinary collaboration, technological integration, and institutional accountability to prevent RSIs. Enhanced postoperative surveillance and heightened clinical suspicion are crucial. This will improve patient safety and uphold healthcare credibility. This case underscores the need for long-term postoperative vigilance, even in the absence of immediate symptoms.

## 1. Introduction

Retained surgical instruments (RSIs) represent a rare but serious complication of surgical procedures, with significant implications for patient safety and healthcare systems [1, 2]. RSIs are defined as surgical items unintentionally left inside a patient's body after surgery, including sponges, sharps, and instruments [3, 4]. Although preventable, RSIs continue to occur, with reported incidence ranging from 1 in 1000 to 1 in 18,000 surgeries [5–7], likely underestimated due to underreporting [8, 9] with mortality associated with RSIs is less frequently quantified but recognized as a serious complication. Commonly affected sites include the abdomen and pelvis [1, 10], with risk factors such as emergency procedures, unplanned procedural changes, high body mass index (BMI), and communication failures among surgical teams contributing significantly [3, 11–13].

Hysterectomy, one of the most frequently performed gynecological surgeries globally, is associated with well-documented complications such as infections, hemorrhage, and organ injury [14–16]. However, the retention of surgical instruments as a postoperative complication remains exceedingly rare and underrecognized [10, 17]. Studies emphasize the role of human error, particularly during manual counts, in the persistence of RSIs despite protocols like the National Safety Standards for Invasive Procedures (NatSSIPs) [4, 11, 18–20]. The lack of consistent implementation and limited use of adjunct technologies such as radiofrequency identification (RFID) and barcoding further exacerbate the issue [21–23].

The consequences of RSIs extend beyond physical morbidity. They often necessitate reoperation, increase healthcare costs, and inflict reputational damage on healthcare institutions [2, 24–26]. The psychological toll on patients, including anxiety and loss of trust in medical providers, is equally significant [2, 27]. This case report details a unique instance of a retained surgical blade discovered 12 years post-hysterectomy, highlighting diagnostic challenges and reinforcing the need for stringent intraoperative reconciliation and vigilant long-term postoperative follow-up [17, 28]. This report is presented in accordance with the SCARE 2023 guidelines [29].

## 2. Case Presentation

A 60-year-old female, Para 7 Living 5, presented with a chief complaint of abdominal pain persisting for 4 years. She reported experiencing generalized abdominal pain throughout the day, colicky in nature, radiating to the back, and temporarily relieved by medication but recurring shortly after. She denied associated symptoms such as nausea, vomiting, fever, changes in bowel habits (constipation or diarrhea), or significant weight loss.

Over the past 4 years, the patient experienced recurrent abdominal pain that led to more than four admissions per year at dispensaries and health facilities across Mwanga and same districts in Moshi, Kilimanjaro. During these visits, she was repeatedly treated for presumed urinary tract infections (UTIs) and prescribed analgesics. In most facilities, she received ciprofloxacin 500 mg twice daily for 5 days, while a few facilities prescribed amoxicillin + clavulanic acid 625 mg twice daily for 5 days. For pain management, she was consistently prescribed paracetamol 1 g three times daily. Despite these repeated treatments, her symptoms persisted, reflecting both misdiagnosis and a significant delay in detecting the retained surgical blade.

Her past surgical history included a total abdominal hysterectomy in 2013 due to heavy bleeding secondary to pedunculated uterine fibroids. She had no history of diabetes mellitus or hypertension. All of her seven children were delivered via spontaneous vaginal delivery, and she entered menopause in 2013 following the hysterectomy.

On general examination, she appeared ill-looking, with a facial expression indicative of pain. However, she was not pale, not jaundiced, and had no generalized lymphadenopathy or lower limb edema.

Her vital signs were as follows: blood pressure (BP) of 138/89 mmHg, pulse rate of 92 beats per minute, respiratory rate of 20 breaths per minute, and temperature of 36.7°C.

Per abdominal examination revealed normal abdominal contours with movement during respiration. A Pfannenstiel incision was noted, along with an inverted umbilicus. Tenderness was elicited on both superficial and deep palpation at epigastric and umbilical region. Percussion revealed a normal tympanic note, and auscultation detected three bowel sounds per minute.

A provisional diagnosis of UTI and peptic ulcer disease was made, with differentials of adhesions, chronic pelvic inflammatory disease, inflammatory bowel disease, and colon tumor.

Laboratory investigations which were done included full blood picture (FBP) which showed normal white blood cell (WBC) of 4.42 × 10^9^/L, hemoglobin (Hb) of 13 g/dL and PLT of 139 × 10^9^/L, RBG of 5.5 mmol/L, urinalysis showed pale yellowish urine with leucocyte 2+, with WBC of 8–15/high power field (HPF); however, there is negative nitrates, proteins, ketones, glucose, and blood.

*H. pylori* testing was performed using the stool antigen method, and the result was negative.

ESR was 5 mm/h (0–10), serum creatinine of 1.14 mg/dL with eGFR of 55 mL/min/1.73m^2^.

**Figure 1:**
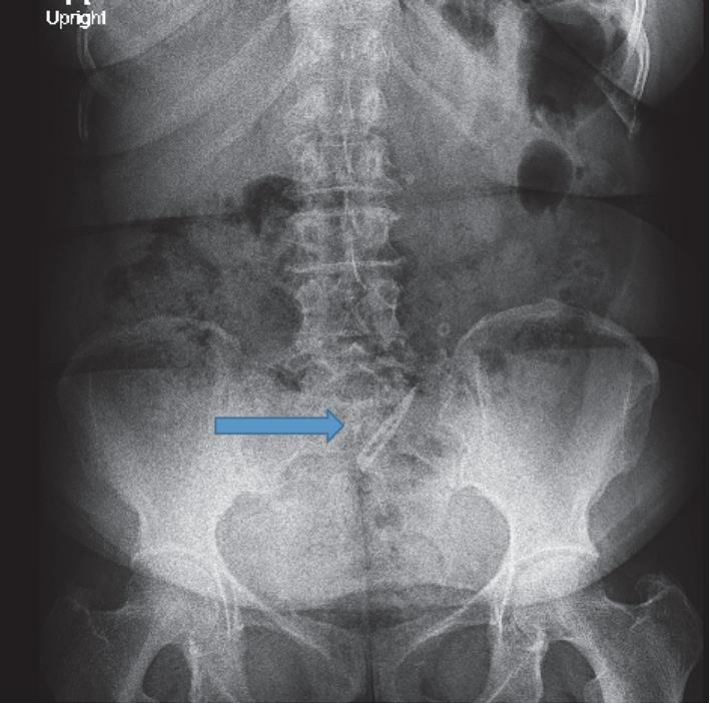
Abdominal X-ray in upright position showing surgical blade in the abdomen.

**Figure 2:**
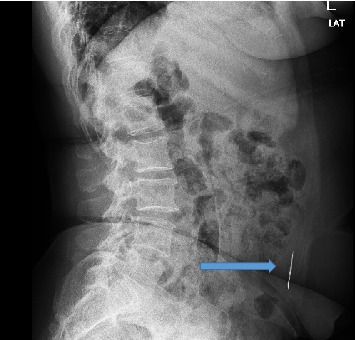
Abdominal X-ray in lateral view showing surgical blade in the abdomen.

**Figure 3:**
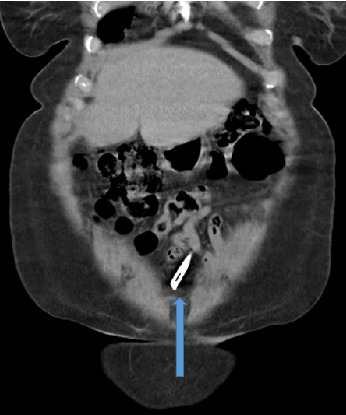
CT scan in coronal view revealed a surgical blade in the abdomen.

**Figure 4:**
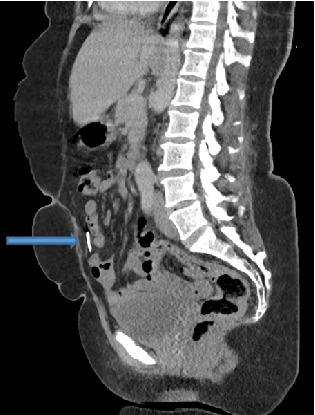
CT scan in sagittal view revealed a surgical blade in the abdomen.

**Figure 5:**
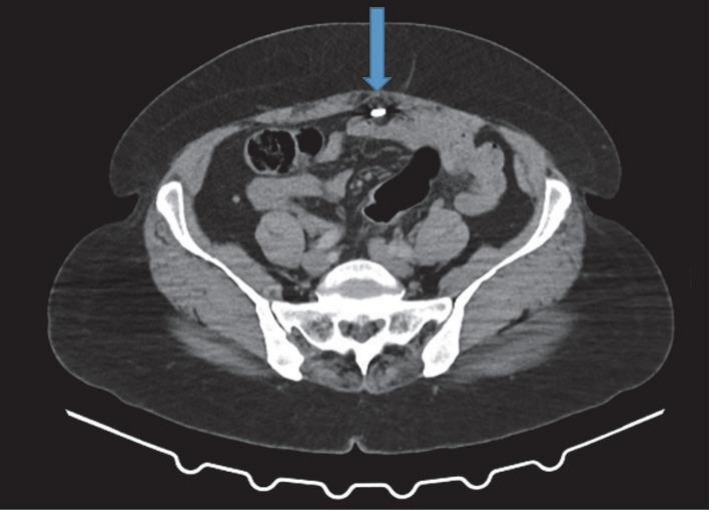
CT scan in axial view revealed a surgical blade in the abdomen.

Abdominal ultrasound showed normal sonographic findings with plain abdominal X-ray showed retained surgical blade as shown in Figures 1 and 2 while computed tomography scan (CT scan) also showed retained surgical blade in different views as illustrated in Figures 3,4 and 5.

**Figure 6:**
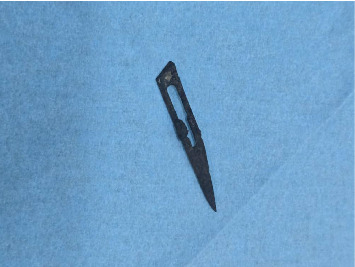
Image showing retained rusted surgical blade number 15 removed from the abdomen.

Elective laparotomy by pfannenstiel incision was done and foreign body was found to be retained rusted surgical blade number 15 as seen in Figure 6 which was rusted and covered by greater omentum located below the anterior abdominal wall 6 cm below the umbilicus. Adhesions release was also done and homeostasis was achieved and incision was closed in layers and patient was returned in the ward.

The patient was then kept on ceftriaxone 1 g bd IV for 5 days and metronidazole 500 mg tds IV for 5 days and pethidine 50 mg tds IM for 2 days and daily wound dressing was done. She was then discharged 5 days later with oral ampicillin + cloxacillin 500 mg tds for 5 days, oral metronidazole 400 mg tds for 5 days, and oral paracetamol 1 g tds for 5 days.

She started weekly follow up to surgical clinic where she made a total of three visits where on her last visit the wound had already closed and the patient was well

## 3. Discussion

The discovery of a retained surgical blade 12 years after a total abdominal hysterectomy exemplifies the enduring patient safety challenge posed by RSIs—designated “never-events” due to their preventable nature [6, 7, 10, 30]. Despite enhanced safety protocols and technological innovations, RSIs persist in both elective and emergency surgeries [3, 8, 18, 31]. This case stands out due to the unusually long latency period and underscores persistent gaps in intraoperative practices and postoperative surveillance.

Nonspecific symptoms such as chronic abdominal pain, misattributed to recurrent UTIs or gastritis, are consistent with previous reports where RSIs elude early detection due to vague presentations [17, 25, 28]. Diagnostic imaging, especially CT scans and plain radiographs, remains a cornerstone for RSI identification [6, 28]; however, the availability of these diagnostics equipments may be challenging due to financial factors as in this case or in limited resource setting. The encapsulation of the blade by omentum likely contributed to delayed symptomatology, as biological tissue often adapts to the foreign body [17].

Though elective, the patient's hysterectomy lacked comprehensive safety measures such as adjunct technologies or enforced surgical counts, which have shown efficacy in reducing RSI incidence [4, 21–23]. Manual counts alone are insufficient, especially during complex procedures or unexpected intraoperative changes [5, 11, 13, 19]. In this case, the most probable culprits were miscommunication among the surgical team and the absence of intraoperative radiography, both of which critically contributed to the retention of the surgical blade. Communication failures among surgical teams have also been implicated in several RSI incidents [2, 27].

RSIs following hysterectomy are rare and often overshadowed by more common complications like vaginal cuff dehiscence or pelvic abscesses [15, 16]. However, retained sharp instruments such as blades pose unique risks, including migration, infection, and fistula formation [1, 17, 32]. Financial implications are considerable; RSI-related lawsuits, extended hospital stays, and revision surgeries create a significant burden on healthcare systems [20, 24, 26].

Preventive strategies must extend beyond conventional counting. Studies advocate for RFID tagging, barcoded surgical sponges, and routine intraoperative imaging in high-risk procedures [21–23]. The effectiveness of these technologies in reducing miscounts and improving surgical outcomes has been well-documented [21, 22]. Institutional policies should also encourage open reporting and systemic learning, addressing the cultural stigma around surgical errors [9, 20, 27].

The patient's prolonged diagnostic journey reflects not only clinical oversight but systemic failure to ensure adequate postoperative follow-up, especially in patients with prior abdominal surgeries [2, 9, 28]. Psychological effects—stress, anxiety, and reduced trust in healthcare—should not be underestimated [2, 27]. As this case demonstrates, a multidisciplinary approach integrating technology, communication, and accountability is essential for mitigating RSI risks and safeguarding patient welfare.

## 4. Conclusion

The case of the retained surgical blade, discovered 12 years post-hysterectomy, underscores the ongoing challenges in preventing retained surgical items. It highlights the need for a multifaceted approach that combines stringent adherence to established protocols, the integration of advanced technologies, and a culture of open communication and continuous learning within surgical teams. Healthcare institutions must prioritize the implementation of standardized counting procedures, explore the use of adjunct technologies like barcoding or RFID, and foster an environment where potential errors are promptly reported and addressed. Hospitals must also adopt mandatory double-check protocols for instrument counts, regardless of surgical complexity.

Furthermore, clinicians should maintain a high index of suspicion for RSIs in patients presenting with unexplained pain or discomfort following surgical procedures, even years after the initial operation. Enhanced postoperative surveillance and robust patient advocacy are essential to minimize the risk of delayed diagnosis and its associated physical and psychological consequences. Ultimately, preventing RSIs requires a collective commitment from surgeons, nurses, and hospital administrators to prioritize patient safety and continuously strive for excellence in surgical care.

## Data Availability

Data sharing is not applicable to this article as no new data were created or analyzed in this study.

## Nomenclature

RSIs: Retained surgical instruments
RFID: Radio-frequency identification
BP: Blood pressure
CT scan: Computed tomography scan
WBCs: White blood cells
Hb: Hemoglobin
HPF: High power field
FBP: Full blood picture.
